# A hybrid feature extraction scheme for efficient malonylation site prediction

**DOI:** 10.1038/s41598-022-08555-9

**Published:** 2022-04-06

**Authors:** Ali Ghanbari Sorkhi, Jamshid Pirgazi, Vahid Ghasemi

**Affiliations:** 1 Department of Computer Engineering, University of Science and Technology of Mazandaran, Behshahr, Iran; 2 Department of Computer Engineering, Faculty of Information Technology, Kermanshah University of Technology, Kermanshah, Iran

**Keywords:** Data mining, Machine learning

## Abstract

Lysine malonylation is one of the most important post-translational modifications (PTMs). It affects the functionality of cells. Malonylation site prediction in proteins can unfold the mechanisms of cellular functionalities. Experimental methods are one of the due prediction approaches. But they are typically costly and time-consuming to implement. Recently, methods based on machine-learning solutions have been proposed to tackle this problem. Such practices have been shown to reduce costs and time complexities and increase accuracy. However, these approaches also have specific shortcomings, including inappropriate feature extraction out of protein sequences, high-dimensional features, and inefficient underlying classifiers. A machine learning-based method is proposed in this paper to cope with these problems. In the proposed approach, seven different features are extracted. Then, the extracted features are combined, ranked based on the Fisher’s score (F-score), and the most efficient ones are selected. Afterward, malonylation sites are predicted using various classifiers. Simulation results show that the proposed method has acceptable performance compared with some state-of-the-art approaches. In addition, the XGBOOST classifier, founded on extracted features such as TFCRF, has a higher prediction rate than the other methods. The codes are publicly available at: https://github.com/jimy2020/Malonylation-site-prediction

## Introduction

Post-translational modification (PTM) is one of the fundamental mechanisms to regulate many biological processes. Today, more than 620 types of PTMs are discovered, including a wide range of chemical groups to a small protein. Malonylation is a recently identified PTM, wherein positively charged lysine amino-acids of a protein are chemically reformed by adding a negatively charged malonyl group, playing a crucial role in various cellular operations, biological processes, and regulating the dynamicity of a cell^1–4^. In 2011, lysine malonylation substrates were identified through proteomic analysis, demonstrating their prominent effects on eukaryote and prokaryote cells^1^. Proteins continuously interact, and incorrect identification of a PTM may result in disease. Therefore, their vigorous and precise scrutiny is needed, through which some daily life mechanisms and conditions, including cancer, diabetes, and auto-immunization, could be identified^5–7^. Regarding the crucial importance of malonylation, precise identification of protein malonylation sites is the primary concern, leading to useful biomedical information and in-depth molecular function perceptions. Thus far, many computational and experimental methods have been proposed for detecting malonylation sites^8^. However, experimental methods suffer from temporal and financial limitations, and their implementations are cumbersome. Hence, an efficient computational method is required to identify the malonylation sites accurately. Some recent works have employed machine learning and deep learning methods to predict malonylation sites^9^. The main contributions of such methods include feature extraction and selection for efficient classification or model representation such as hybrid or deep learning models.

In^10^, the “Mal-Lys” method is presented to predict K-mal sites. In this approach, residue sequence order information, position-specific amino acid propensity, and physicochemical properties are extracted as features. Then, the significant features are identified by the *“minimum redundancy maximum replication” (mRMR) approach.* Eventually, the existence of a malonylation site is predicted via a support vector machine (SVM). Wang et al.^11^ proposed a novel method for malonylation site recognition based on unique sequences, evolutionary profiles of sequences, and amino-acid attributes. In^12^, sequence orders, gene ontologies, and their composition have been used as features, and an SVM is used for classification. The result has shown that feature combination yields more efficient results. In the “SPRINT Mal” method^13^, some ordinal and structural features are extracted out of the protein sequences. It is the first online prediction scheme that has pondered the structural attributes of proteins. The prediction is carried out by an SVM too.

In^14^, a variety of 11 features is extracted out of protein sequences. Regarding the high-dimensionality of the feature vectors, the features are further processed by their gain ratio, and the significant features are selected. Then, several classifiers are employed, such as a decision tree, support vector machine, K-nearest neighbors, logistic regression, and light gradient boosting machine. In^15^, the features are extracted regarding the neighboring amino-acid interactions using a B-peptide-based scheme. Then, the *light gradient boosting classification* is incorporated to identify the malonylation sites.

In^16^, pseudo-amino acids have been used as features to train an SVM classifier to identify malonylation sites. In^17^, a novel approach, called CKSAAP_FormSite, is proposed. In this method, an efficient feature extraction scheme based on the composition of k-spaced acid pairs is used for encrypting malonylation sites. Then, malonylation sites are detected using SVM. In^18^, a 3-phase approach is presented. Features are extracted based on sequence orders in the first stage. Then, the data of both classes are balanced using random sampling. Eventually, malonylation sites are predicted by a random forest classifier.

In^19^, a machine learning-based scheme is proposed for predicting malonylation sites. In this approach, physicochemical attributes, sequential, structural, and functional information of proteins are used as features. Then, mRMR and *symmetrical uncertainty* methods were used for efficient feature selection. The classification model is SVM. Feature composition is considered in^20^. In this scheme, one-hot coding, physicochemical attributes, and composition of k-spaced acid pairs are considered for feature extraction. Then, *principal component analysis (PCA)* is used to extract efficient features, and an SVM is used to predict malonylation sites. In^21^, amino acid’s predicted secondary structure is used to extract two types of structural features out of neighboring amino acids on protein sequences. The results show that the proposed method has a promising performance.

Recently, deep learning-based approaches have gained ground for predicting malonylation sites. However, these methods are not end-to-end and need to extract features from the input data. The extracted features are fed into the deep networks. Moreover, a great deal of training data is required to tune the parameters of the deep networks, while a short amount of data is confronted yet. In^22^, a hybrid model, including a convolutional neural network (CNN) and the composition of physicochemical attributes, evolutional information, and sequential features, is used to identify mammals’ protein malonylation sites.

In^23^, a deep learning (DL) model is proposed based on *long-short term memory (LSTM)* together with word embedding for malonylation sites prediction. The proposed method outperforms the traditional approaches using various extracted features and LSTM-based DL classification with a one-hot vector. This method suffers from being sensitive to the size of the training set; however, a concoction with traditional machine learning may overcome the weakness. In^24^, *conditional general adversarial networks (CGAN)* have been used to identify seven different types of malonylation sites. Primarily, the features are extracted via eight different sequential and four structural feature extraction schemes. Then, the number of features is augmented to 1479 using Pearson correlation. Afterward, both classes’ instances are balanced by a CGAN and a *Conditional Wasserstein Generative Adversarial Network (CWGAN)*. A random forest classifier is incorporated to predict malonylation sites.

In^25^, a *multi-layer perceptron (MLP)* is presented. In this approach, six different features are extracted from protein sequences, and an MLP is hired for malonylation site prediction. A DL-based method is presented in^26^ to increase the prediction rate. For this purpose, some features such as position-specific amino acid composition, the composition of k-spaced acid pairs, and position-specific scoring matrix are extracted from protein sequences. Then, maximal dependence decomposition is hired to extract efficient features. Eventually, a multi-layered DL network carries out the classification. Transfer learning approaches have been incorporated to achieve prediction on large scales in^27^. In this work, a recurrent neural network-based deep learning model is primarily trained and then tuned using propionylation. The trained model is used for feature extraction, such that it is fed with a protein sequence and yields the due feature vector as output. An SVM is used for the final classification. In^28^, five different feature types are extracted from protein sequences. A feature vector of length 1431 ensues. The resulted features are fed into a CNN. The classification is carried out in the last layers, which are fully connected.

In^29^, DeePPSite is presented for phosphorylation site prediction based on LSTM neural networks. In this method, various features, including PSSM, IPC, and EGBW, are extracted. The prediction is then carried out via LSTM. In^30^, the site prediction is conducted by hot-encoder feature extraction and CNN classifiers. In this approach, the features are extracted via the hot-encoder method. The extracted features are then fed into a one-dimensional CNN classifier. In^31^, various feature sequences are used for malonylation site prediction. The prediction is carried out based on DNNs. A method called NearMiss-2 is used in this approach to cope with imbalanced data. In^32^, eight different feature extraction schemes and three structural features have been studied. In this approach, various features are combined, and the performance is higher tone-dimensional features.

The primary focus of the present work is on delivering a novel feature extraction strategy to predict the malonylation sites efficiently. For this purpose, primarily, various features are extracted out of protein sequences. The primary features are combined, each combination is assessed, then weighed, and the best one is selected. Features are selected based on the Fisher’s score (F-score) to select efficient features and avoid model over-fitting. Eventually, the classification is carried out via various classifiers, including random forest (RF), extreme gradient boosting (XGBoost), SVM, and DNN. Totally, a five-stage approach is proposed in the present work, in which the feature extraction is carried out in the first stage. A preprocessing of the extracted features is conducted in the second stage. The third stage is dedicated to selecting features out of various combinations. Eventually, a classification is achieved in the fourth stage to predict malonylation sites. The model assessment is carried out at stage five. Specific contributions and novelties of this paper can be summarized as follows:

- The *term frequency and category relevancy factor (TFCRF)* method for weighting features is investigated. Some weighting schemes inspired by document analysis have already been used for malonylation site prediction; however, to the best of our knowledge, TFRCF has not been explored yet. In this method, the distribution of features within various classes is considered along with their distribution in entire sequences of all classes. The results show the efficiency of TFCRF.
- The proposed feature combination scheme provides a feature-level diversity, improving amino-acid sequence classification. That is, each combined feature includes a specific piece of information. TFCRF feature includes binary classification distribution information, *position-specific scoring matrix (PSSM)* contains genomic sequence information and other features envelope frequency information. This strategy has been seldom investigated in the related works thus far.
- Selecting relevant features and omitting redundant ones is another novelty of the proposed method, which has rarely been considered in previous works. For this purpose, the best feature combination is selected based on Fisher’s score.

The remaining sections of the paper are as follows. Section “Feature extraction” describes various feature extraction schemes for malonylation site identification. Section “The proposed method” elaborates the five stages of the proposed method, including feature extraction, preprocessing, feature selection, classification, and model assessment. Section “Experimental results” describes the experimental results for the proposed approach, and the outcomes are compared with several other common methods. Finally, Section “Conclusion” concludes the paper.

## Feature extraction

One of the most important phases in malonylation site prediction is feature extraction. A primary approach is to extract various pre-known features out of protein sequences, and then, a classification process is devised. A secondary approach is to design an end-to-end deep neural network model, through which significant features can be extracted systematically, and the classification could be conducted upon the basis of such features. No end-to-end model has been proposed for the secondary approach thus far. In most of the presented works, the features are extracted using known feature extraction methods, and then classical machine learning or deep learning models are incorporated for classification. Typically, end-to-end models are not recommended due to the lack and insufficient data for training plenty of parameters in deep neural networks. So, we opt to extract significant pre-known features out of protein sequences in the proposed method. The sequential nominal character information can be converted to a numerical vector using several feature extraction algorithms. Extracting efficient features will enhance the performance of the classification. To extract features out of protein sequences the following algorithms are incorporated: *the enhanced amino acid composition (EAAC)*^33^ *the enhanced grouped amino acid composition (EGAAC)*^33^, *dipeptide deviation from expected mean (DDE)*^34^, *PKA*^35^, *term frequency-inverse document frequency (TFIDF)*^36^, *TF_CRF*^37^, and *position-specific scoring matrix (PSSM)*^38^. These methods are elaborated in the following subsections.

### Enhanced amino acid composition (EAAC)

This method is presented by Chen et al.^33^. In this algorithm, sequential protein information is extracted, and accordingly, amino-acid frequency information is calculated as^33^:

1 $$g\left(m,n\right)=\frac{H\left(m,n\right)}{H\left(n\right)} , m\in \left\{A,C,D, \cdots ,Y\right\} ,n\in \left\{W1,W2,\cdots WL\right\}$$

where $H(m,n)$ is the number of amino-acid type $m$, and $H(n)$ is the length of the $n$’th window length.

### Enhanced grouped amino acid composition (EGAAC)

In this method, protein sequences are converted to numerical feature vectors based on their attributes. It is a compelling feature extraction algorithm in bioinformatics research fields such as malonylation site prediction.

EGAAC is computed based on amino-acid categorization. In^39^, amino acids are categorized based on five physicochemical characteristics: aliphatic (including GAVLMI amino-acids), aromatic (including GFYW amino-acids), positively charged (including KRH amino-acids), negatively charged (including DE amino-acids), and neutral or uncharged (including STCPNQ amino-acids). Accordingly, EGAAC is calculated based on the following equation:

2 $$G\left(g,n\right)=\frac{H\left(g,n\right)}{H\left(n\right)} , g\in \left\{g1,g2,g3,g4,g5\right\} , n\in \left\{W1,W2,\cdots WL\right\}$$

where $g$ is one of the five categories, $H(g,n)$ is the number of amino acids in group $g$, and $H(n)$ is the length of $n$’th window^33^. A window size of length five is considered in this paper.

### Dipeptide deviation from expected mean

Dipeptide deviation from the expected mean (DDE) is proposed and developed in^34^, wherein feature extraction based on amino-acid combination is studied to discriminate a cell’s epitopes and non-epitopes. For this purpose, the dipeptide combination (DC) of a protein sequence is primarily calculated as:

3 $$DC\left(m,n\right)= \frac{{H}_{mn}}{H-1}  ,  m,n\in \left\{A,C,D,\cdots ,Y\right\}$$

where ${H}_{mn}$ is the number of paired $mn$ amino-acids, and H is the size of the protein sequence. Next, a protein’s theoretical mean (TM) and theoretical variance (TV) are computed as:

4 $$TM\left(m,n\right)= \frac{{C}_{m}}{{C}_{H}}\times \frac{{C}_{n}}{{C}_{H}}$$

5 $$TV\left(m,n\right)= \frac{TM\left(m,n\right)\left(1-TM\left(m,n\right)\right)}{H-1}$$

where ${C}_{m}$ and ${C}_{n}$ are the number of codons encrypting the first and the second amino-acids, respectively, and ${C}_{H}$ is the total number of codons. Finally, DDE is calculated based on TV, TM, and DC as:

6 $$DDE\left(m,n\right)= \frac{DC\left(m,n\right)\left(1-TM\left(m,n\right)\right)}{\sqrt{TV\left(m,n\right)}}$$

### PKA

This feature is the negative logarithm of the isolation constant for every group in the molecule ^35^.

### Term frequency: inverse document frequency

TF_IDF feature extraction is composed of two terms, TF and IDF, which stand for the *term frequency* and *inverse document frequency*, respectively. Both terms should be calculated separately and multiplied to yield the TF_IDF coefficient^36^. Each term is defined as follows:

$TF(t,d)$: the number of amino-acid $t$ in a protein sequence, divided by the size of the protein, namely $d$.

$IDF(t)$: the logarithm of the total number of proteins (namely $\left|D\right|$) divided by the number of contents which include amino-acid $t$ (namely $DF(t)$). It is calculated as:

7 $$IDF\left(t\right)= \mathrm{log}\left(\frac{\left|D\right|}{DF\left(t\right)}\right)$$

Having calculated TF and IDF, TF-IDF is calculated as:

8 $$TF-IDF\left(t,d\right)=TF\left(t,d\right)\times IDF\left(t\right)$$

### Term frequency and category relevancy factor (TF-CRF)

In this method, two factors, namely positiveRF (positive relation frequency) and negativeRF (negative relation frequency), are defined as follows^37^:

#### PositiveRF

This factor is the ratio of the number of amino acids in a protein sequence ${c}_{i}$, having a common characteristic ${t}_{k}$, to the total number of amino acids in the protein sequence. It is calculated as:

9 $$PosotiveRF\left({t}_{k},{c}_{{d}_{i}}\right)=\frac{\left|D\left({t}_{k},{c}_{j}\right)\right|}{\left|D\left({c}_{j}\right)\right|}$$

#### NegativeRF

This factor is the ratio of the total number of amino acids in protein sequences except for ${c}_{i}$, having a common characteristic ${t}_{k}$, to the total number of amino acids in protein sequences except for ${c}_{i}$. It is calculated as:

10 $$NegativeRF\left({t}_{k},{c}_{{d}_{i}}\right)= \frac{\sum_{m=1 ,m\ne j}^{\left|c\right|}\left|D\left({t}_{k},{c}_{m}\right)\right|}{\sum_{m=1 ,m\ne j}^{\left|c\right|}\left|D\left({c}_{m}\right)\right|}$$

where $\left|D({c}_{j})\right|$ is the number of amino acids in protein sequence ${c}_{j}$, and $\left|D({t}_{k},{c}_{j})\right|$ is the number of amino acids in the set $D$ and protein ${c}_{j}$ with common characteristic ${t}_{k}$.

*Category relevancy factor value (crfValue)* is defined as follows, considering the equations mentioned above:

11 $$crfValue({t}_{k},{c}_{j})=\frac{PosotiveRF\left({t}_{k},{c}_{j}\right)}{NegativeRF\left({t}_{k},{c}_{j}\right)}$$

The relevance factor of each category has a direct relation with positiveRF and a reverse relationship with negativeRF. Accordingly, the proposed weighting for feature ${t}_{k}$ in protein sequence $d_{i}$ is:

12 $$w_{ki} = \log \left( {tf\left( {t_{k} ,d_{i} } \right) \times crfValue\left( {t_{k} ,c_{{d_{i} }} } \right)} \right) = \log \left( {tf\left( {t_{k} ,d_{i} } \right) \times \frac{{PosotiveRF\left( {t_{k} ,c_{{d_{i} }} } \right)}}{{NegativeRF\left( {t_{k} ,c_{{d_{i} }} } \right)}}} \right)$$

where ${c}_{{d}_{i}}$ is the category of protein sequence ${d}_{i}$. Normalization is used to mitigate the effect of the length of the sequence on the classification performance. It confines the weights in the range $(\mathrm{0,1})$. The final equation of TFCRF will be:

13 $$W_{ki} = TFCRF\left( {t_{k} ,d_{i} } \right) = \frac{{\log (tf\left( {t_{k} ,d_{i} } \right) \times crfValue\left( {t_{k} ,c_{{d_{i} }} } \right))}}{{\sqrt {\mathop \sum \nolimits_{k} (\log (tf\left( {t_{k} ,d_{i} } \right) \times crfValue\left( {t_{k} ,c_{{d_{i} }} } \right)))^{2} } }}$$

Accordingly, the content of each protein sequence is represented by a feature vector ${d}_{i}=({W}_{1i},{W}_{2i},\dots ,{W}_{ki})$, where $k$ is the total number of selected features, and ${w}_{ki}$ is the weight of feature (i.e., amino-acid) ${t}_{k}$ in sequence ${d}_{i}$. ${W}_{ki}$ indicates to what extent feature ${t}_{k}$ includes the concept of protein sequence ${d}_{i}$.

Most class-based weighting methods, such as IDF, have been used for information retrieval (IR) and document analysis purposes. These methods have not been applied in protein sequence classification. Hence, some aspects of IR and document analysis, also associated with protein sequence classification, have been neglected. The weighting method of TFCRF contains such elements, as stated in the following.

**Figure 1:**
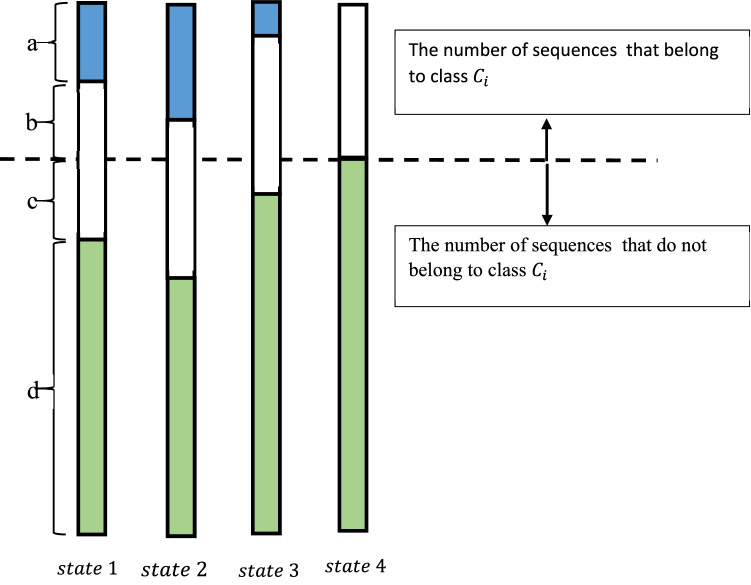
Distributions of feature $x$ across classes in 4 hypothetical states.

Consider a set of protein sequences that belong to a number of classes, with a specific number of instances. Figure 1 depicts various distributions of a feature, namely $x$, in 4 hypothetical states regarding a class, namely ${c}_{i}$. In this figure, $a$ and $b$ are the numbers of sequences in class ${c}_{i}$ that include and exclude feature $x$, respectively; also, $c$ and $d$ denote the number of sequences in all classes other than ${c}_{i}$ that include and exclude feature $x$, respectively. The frequency of feature $x$ is taken constant in all states.

In IDF-based schemes, the weight of every feature is inversely related to the number of sequences including that feature. In the above instance, the weight of feature $x$ in class ${c}_{i}$ can be calculated via (7) as:

14 $$idf\left(x\right)=\mathrm{log}\frac{N}{b+c}=\mathrm{log}\frac{a+b+c+d}{b+c}$$

where $N$ is the total number of sequences. Lets ${w}_{x}^{s}$ denote the weight of feature $x$ in state $s$ of Fig. 1. Then, the relation between the weights of $x$ in various states will be:

$${w}_{x}^{1}={w}_{x}^{2}={w}_{x}^{3}={w}_{x}^{4}$$

As it can be seen, the weight of feature $x$ will not change in various states due to the identical number of sequences including it (i.e., $b+c$); while the status of feature $x$ is apparently changed in class ${c}_{i}$ in multiple states, and this fact is overlooked in weighting this feature. Furthermore, in IDF-based approaches, the more the number of sequences including a specific feature, the less discrimination the feature will have; hence, it is assigned a lower weight. Although this is an accurate hypothesis in IR, it needs to be reformed for the purpose of protein classification. As evident from Fig. 1, despite a significant number of sequences including $x$, if most of those sequences belong to the same class ${c}_{i}$ (cases 3 and 4 in Fig. 1), feature $x$ is not only efficient, but also it must be known significant to discriminate class ${c}_{i}$ from others and dedicated a great weight. In addition, a lower weight should be dedicated to $x$ in class ${c}_{i}$ if a great number of sequences of classes other than ${c}_{i}$ include feature $x$ (state 2 in Fig. 1).

The introduced crfValue in TFCRF delivers a solution for the abovementioned problem. That is, the weight of every feature in each sequence has a direct relation with the number of sequences belonging to the class of that sequence and an inverse relation with the number of sequences belonging to the other classes. In the presented example of Fig. 1, the weight of feature $x$ in class ${c}_{i}$ via (11) equals:

15 $$crfValue(x,{c}_{i})=\frac{\frac{b}{b+a}}{\frac{c}{c+d}}$$

As a result, the relation between weights feature $x$ in Fig. 1 will be:

$${w}_{x}^{2}<{w}_{x}^{1}<{w}_{x}^{3}<{w}_{x}^{4}$$

It can be seen that in this method, the effect of classes, in which the features attend, is taken into account. It should be noted that crf Value is not independent of the number of sequences in each class, drastically increasing the performance of sequence classifiers.

### PSSM

Position-specific scoring matrix (PSSM) is a scoring matrix used in the protein BLAST search, in which a score is dedicated to each amino acid separately, based on its position in the sequence of a number of proteins^41^. This matrix can be shown as:

16 $$PSSM=\left[\begin{array}{ccc}{P}_{1,1 }& \cdots & {P}_{1,20}\\ \vdots & \ddots & \vdots \\ {p}_{L,1}& \cdots & {P}_{L,20}\end{array}\right]$$

where $L$ is the protein sequence length with a number of 20 possible amino acids. Each element of the PSSM matrix is calculated as:

17 $${P}_{i,j}={\mathrm{log}}_{2}\left(\frac{{M}_{i,j}}{{b}_{j}}\right)$$

where ${M}_{i,j}$ is the probability of amino-acid $j$ attending at position $i$, and ${b}_{j}$ is the background model for amino-acid $j$ (e.g. ${b}_{j}=0.05$ by postulating a uniform distribution for amino acids). PSSM scores are positive or negative values. Positive values show that the due amino-acid locational presence occurs more than expected stochastically, while the negative values depict that it takes place less than what is anticipated. PSSM includes locational and evolutionary information of protein sequences.

## The proposed method

**Figure 2:**
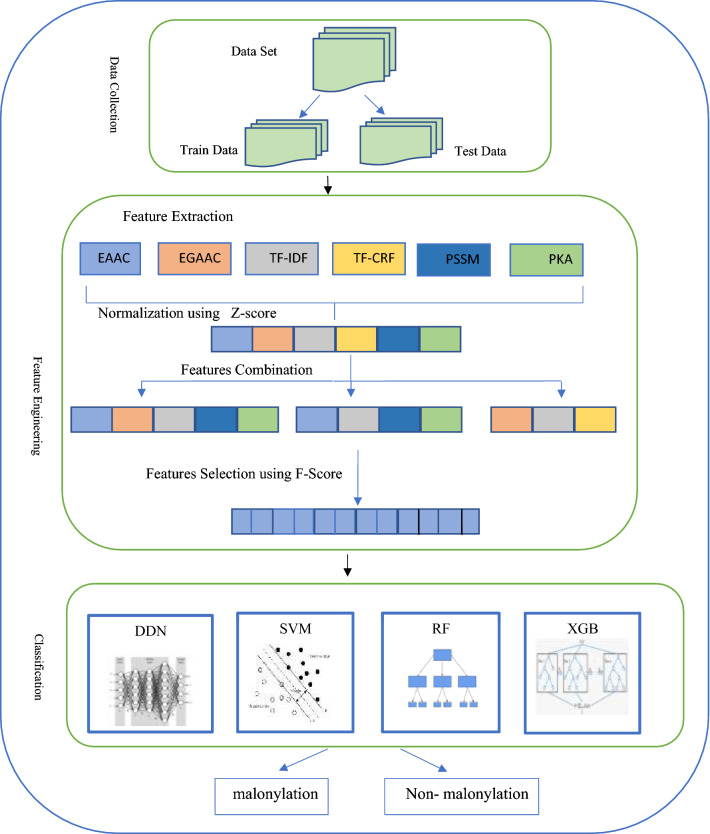
The overall block diagram of the proposed method.

This section proposes a novel model for predicting malonylation sites based on feature extraction and machine learning algorithms. The overall schema of the proposed method is depicted in Fig. 2. It comprises five major stages: dataset selection, feature extraction, feature normalization, feature selection, and classification. Each stage is elaborated in the following.

### Stage 1: dataset selection

Three datasets, namely *Escherichia coli*, *Mus musculus*, and *Homo sapiens*^40^, have been hired for training and testing the proposed method. The dataset is randomly divided into train and test sets. For efficient analysis, a tenfold cross-validation strategy is conducted. At each iteration, one fold is used as a test set, and the remaining nine folds are incorporated for training the model. Model parameters are tuned based on the training sets. The ultimate result is the average results of 10 iterations.

### Stage 2: feature extraction

At this stage, feature extraction methods including EAAC, EGAAC, TFIDF, PSSM, and TF-CRF have been applied as:

- *EAAC and EGAAC* in EAAC, amino-acid frequencies are calculated, and in EGAAC, the protein sequences are converted to numerical vectors based on their characteristics. The resulting feature vectors will be of lengths 20 and 45, respectively.
- *TF-IDF* it is used for calculating the weighted frequency of amino acids. This method shows the frequency of amino acids and aims to depict an amino acid’s significance by comparing its frequency in the dataset with a larger reference dataset. The resulting feature vector will be of length 20 in this method.
- *TF-CRF* it is used for more precise weighting by two factors, i.e., psitiveRF and negativeRF. The resulting feature vector is of length 20.
- *PSSM* a score is dedicated to a selected amino acid, solely based on its location in a protein sequence. The resulting feature vector will be of length 400.
- *PKA* includes negative logarithm of isolation for each group in a molecule. The values pertaining to each amino acid are taken into account. The result will be a single numerical feature.

### Stage 3: preprocessing

Having extracted features out of protein sequences, they would be of various ranges. The difference in feature values would plummet the effect of some important features. In the present work, the primitive values of features range from 0 to 0.03 and, in some cases, from 0 to 200. Additionally, hiring features with a sprawling domain of fluctuations deteriorate the efficacy of the underlying learning models. Accordingly, the data should be normalized to improve efficiency. In the present work, Z-score normalization is used for this purpose.

In fact, Z-score is a normalization strategy that prevents outlier data and features. The normalization equation is as follows.

18 $$z=\frac{x-\upmu }{\upsigma }$$

where $\mu$ and $\sigma$ are mean and variance of feature $x$. If a value equals the mean, it is normalized to zero. If it is less or greater than the mean, it is normalized to a negative or positive value. The magnitude of this negative/positive value is determined based on the variance. The variance of an abnormal feature would be a large number, and its normalized values dwindle to zero.

### Stage 4: feature selection

The extracted features are used for malonylation site prediction. However, all of the features may not be efficient. Some of them may be irrelevant, and some may be redundant. Such features results in model overfitting. Therefore, it is needed to preserve relevant features. Fisher’s score (F-score) method, a filter-based approach, is applied to identify relevant features. F-score criteria for the $i$’th feature is calculated as:

19 $$F-Score\left(i\right)= \frac{\sum_{k=1}^{m}{{n}^{k}\left({\overline{x} }_{i}^{k}- {\overline{x} }_{i}\right)}^{2}}{\sum_{k=1}^{m}\frac{1}{{n}^{k}-1}\sum_{j=1}^{{n}^{k}}{\left({x}_{j,i}^{k}- {\overline{x} }_{i}^{k}\right)}^{2}}$$

where ${\overline{x} }_{i}^{k}$ and ${\overline{x} }_{i}$ are the mean of the $i$’th feature in the class $k$ and the whole dataset, respectively, ${x}_{j,i}^{k}$ is the $i$’th feature value of instance $j$ in class $k$, ${n}^{k}$ is the number of instances in class $k$, and $m$ is the total number of classes. A number of highly-ranked features are selected for classification in the next stage.

The key idea of the Fisher score is to find a subset of features, such that in the data space spanned by the selected features, the inter-class distances of data points are maximized while the intra-class distances are minimized. Since this is a combinatorial optimization problem, it is reduced to computing a score for individual features, independently, via the scoring function of (19); then, a number of highly-ranked features are selected. In (19), the nominator and denominator represent inter-class and intra-class distances, only with regard to feature ${x}_{i}$, respectively. Although some informative dependencies between features are ignored, this method will reduce the time complexity of feature selection to a linear order.

### Stage 5: model assessment

A tenfold class validation strategy is conducted to assess the prediction performance of the classification model. The classifiers include XGBoost, SVM, RF, and DNN. Various measures, including AUC, ACC, Sn, Sp, and MCC, have been used for performance assessment.

## Experimental results

### The datasets

A pilot confirmed dataset is hired for the simulations^40^. The dataset includes 1746 malonylation sites of 595 proteins in “*E. coli*”, 3435 malonylation sites of 1174 proteins in “*M. musculus*”, and 4579 malonylation sites of 1660 proteins in “*H. sapiens*”^40^. The length of amino-acid sequences is reduced to 25, centered at lysine (K). Table 1 elaborates the characteristics of the dataset.

**Table 1:** The number of malonylation and non-malonylation samples in the dataset.

Dataset	Species	Number of malonylation samples	Number of non-malonylation samples
Training set	*E. coli*	1453	1453
	*H. sapiens*	3585	3585
	*M. muscuus*	2606	2606
Independent test	*E. coli*	100	100
	*H. sapiens*	300	300
	*M. muscuus*	600	600

### Model assessment

A tenfold cross-validation strategy is conducted to tune the models’ parameters based on the training dataset, and the independent set is used for testing the model. Efficiency measures *sensitivity (sn)*, *Specificity(Sp)*, *accuracy (acc)*, and *Mathew’s correlation coefficient* (MCC) have been used to assess the underlying models^42^. These measures are calculated as follows.

20 $$Sn=\frac{TP}{TP+FN}$$

21 $$Sp=\frac{TN}{TN+FP}$$

22 $$ACC=\frac{TP+TN}{TP+TN+FP+FN}$$

23 $$MCC=\frac{TP\times TN-FP\times FN}{\sqrt{(TP+FN)(TP+FP)(TN+FP)(TN+FN)}}$$

where TP, TN, FP, and FN denote the number of true positives, true negatives, false positives, and false negatives, respectively.

### Sequence analysis

**Figure 3:**
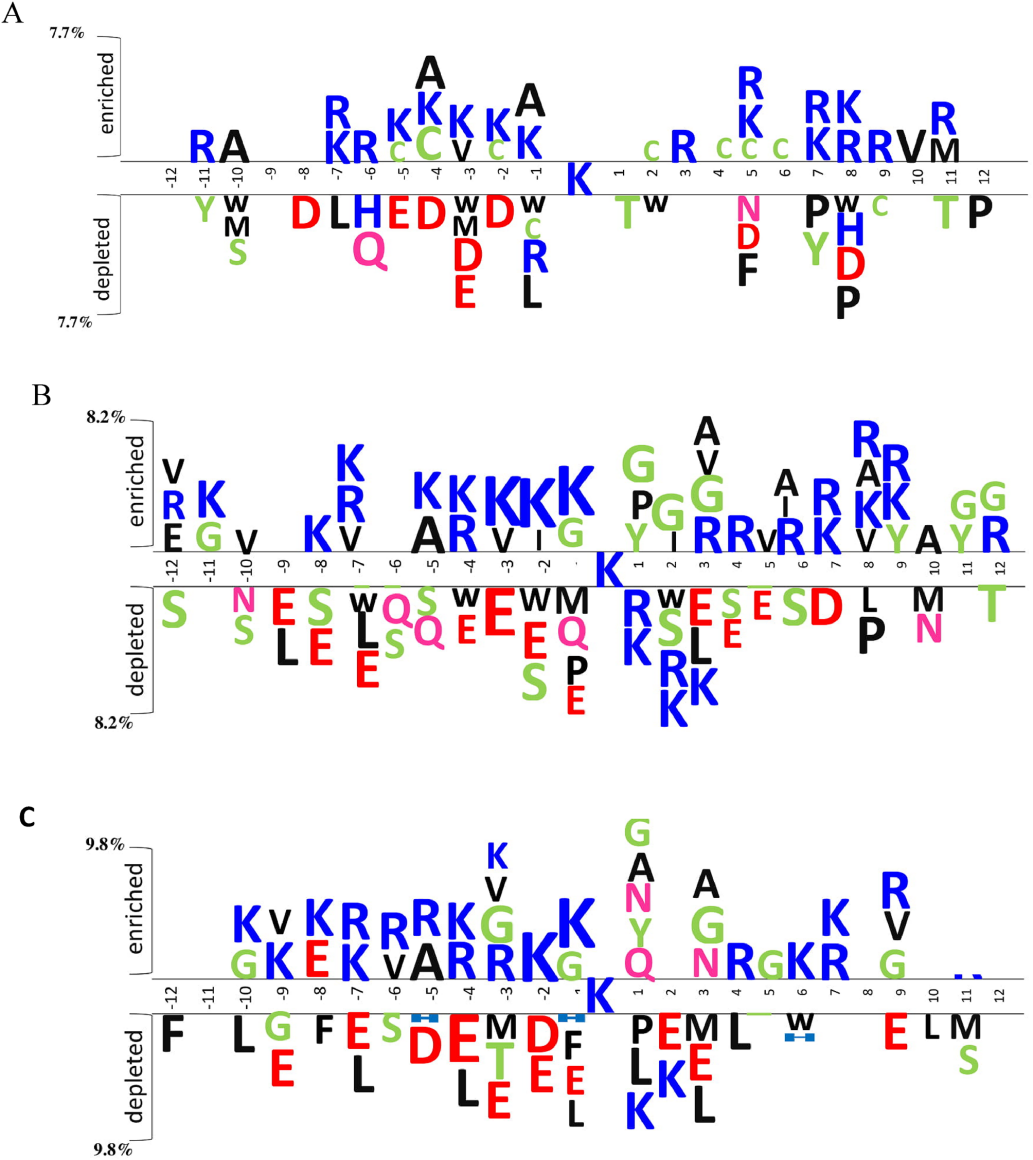
The distribution of amino acids around the central lysine in (**A**) *E. coli*, (**B**) *H. sapiens* and (**C**), and *M. musculus* datasets.

The datasets of “*H. sapiens*,” “*E. coli*,” and “*M. musculus*” have been incorporated to discriminate malonylation and non-malonylation sites. The statistical differences between protein sequences of malonylation and non-malonylation sites in the datasets mentioned above are depicted in Fig. 3^28^. This figure represents the amino-acid distribution of a protein sequence in the dataset. As shown, lysine is located at the center, and the significantly enriched/depleted surrounding residues are described in the range − 12 to + 12. The diagram depicts a significant difference in amino-acid frequencies between protein sequences of malonylation and non-malonylation sites in various sequence fragments. Compared to central lysine, an arbitrary amino acid is studied in two sections, i.e., *enriched* and *depleted*. It is observed that the frequency of amino acids is higher around central lysine than the other fragments in the enriched section. The more distant from the central lysine, the less frequency is observed. Moreover, the exclusive enriched/depleted amino acids around the central lysine unfold the importance of feature selection based on ordinal protein sequences. Accordingly, the importance of a feature extraction scheme based on the combination of multiple sequential features comes into the light to predict the malonylation sites more efficiently.

### Feature extraction analysis

As described earlier, it is sought to extract different features out of protein sequences in order to identify malonylation sites precisely. In this study, seven feature extraction schemes were applied to protein sequences. A random forest classifier was trained based on each feature scheme EAAC, EGAAC, PKA, DDE, TF-IDF, TF-CRF, and PSSM through a tenfold cross-validation strategy to assess the attributes of each method.

**Figure 4:**
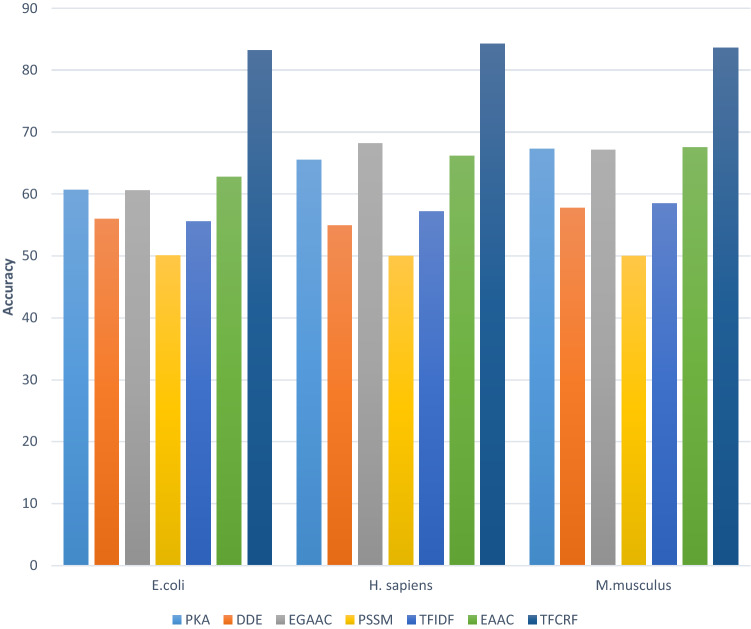
Classifiers’ performance comparison, based on singular features.

The results are depicted in Fig. 4 for the three datasets. It is observed that TF-CRF is more discriminative than the others, with higher accuracy in all of the datasets. Moreover, EAAC, EGAAC, and PKA have promising and comparable results. Based on these results, the combination of features was exploited, and the RF classifier was trained and tested by each combination. In order to obtain the best features, they have been combined and compared with each other. In this phase, the features are selected and combined randomly. The features with higher independent prediction rates have been of higher selection priority. At this stage, combinations of 2 to 5 features have been assessed and compared with each other, primarily.

Three combinations outperformed the others: (1) the combination of TF-CRF, EGAAC, and TF-IDF with a vector of 228 features, (2) the combination of EAAC, PKA, PSSM, and TF-CRF, with a vector of 494 features, (3) the combination of EAAC, PKA, PSSM, TF-CRF, and EGAAC with a vector of 599 features. The results of incorporating these feature combinations into various classification models and the ensued performance measures are taken in Table 2.

**Table 2:** The performance of classifiers with various feature combinations.

Dataset	Combination	Classifier	Acc (%)	Sn (%)	Sp (%)	MCC	AUC
*E. coli*	TF-CRF, EGAAC, TF-IDF	SVM	59.33	60.91	57.75	0.6839	0.7223
		RF	91.44	93.45	93.78	0.8902	0.9695
		XGBoost	95.18	93.03	95.21	0.9049	0.981
		DNN	68.63	86.09	51.14	0.7348	0.7891
	EAAC,PKA, PSSM, TF-CRF	SVM	67.03	68.34	65.73	0.8261	0.8834
		RF	96.69	93.59	95.79	0.9151	0.9711
		XGBoost	97.21	94.31	95.72	0.9279	0.9768
		DNN	92.99	92.46	93.52	0.9023	0.9649
	EAAC, PKA, PSSM, TF-CRF, EGAAC	SVM	66.83	68.83	64.84	0.8311	0.8714
		RF	96.69	93.27	95.38	0.9147	0.9723
		XGBoost	**97.22**	**94.64**	**95.78**	**0.9311**	**0.9781**
		DNN	90.10	99.25	80.92	0.8973	0.9578
*H. sapiens*	TF-CRF, EGAAC, TF-IDF	SVM	65.90	66.42	65.38	0.7482	0.8831
		RF	92.15	90.88	93.42	0.9087	0.9489
		XGBoost	94.18	96.18	95.08	0.9234	0.9634
		DNN	77.99	87.75	68.22	0.7841	0.9043
	EAAC,PKA, PSSM, TF-CRF	SVM	69.86	68.67	71.04	0.8418	0.8931
		RF	93.24	91.76	94.02	0.9287	0.9528
		XGBoost	**95.22**	**97.32**	**97.14**	**0.9448**	**0.9749**
		DNN	91.17	94.36	94.89	0.9142	0.9328
	EAAC, PKA, PSSM, TF-CRF, EGAAC	SVM	68.66	67.87	69.45	0.8346	0.8911
		RF	92.73	91.24	93.61	0.8971	0.9518
		XGBoost	94.71	97.1	96.42	0.9371	0.9659
		DNN	91.45	93.45	92.61	0.9017	0.9503
*M. muculus*	TF-CRF, EGAAC, TF-IDF	SVM	64.37	65.16	63.58	0.7934	0.8942
		RF	91.46	91.90	93.02	0.8872	0.9537
		XGBoost	92.88	95.23	93.78	0.8943	0.9644
		DNN	72.85	89.14	56.56	0.8136	0.8993
	EAAC,PKA, PSSM, TF-CRF	SVM	71.74	70.91	72.56	0.8623	0.9061
		RF	92.76	93.23	94.48	0.9023	0.9573
		XGBoost	**94.31**	**96.47**	**95.34**	**0.9217**	**0.9721**
		DNN	90.87	92.43	94.09	0.8983	0.9382
	EAAC, PKA, PSSM, TF-CRF, EGAAC	SVM	70.95	70.49	71.41	0.8582	0.9035
		RF	92.21	93.82	93.47	0.8991	0.9548
		XGBoost	93.29	95.93	94.62	0.915	0.9692
		DNN	91.15	93.72	94.56	0.8932	0.9376

Significant values are in bold.

In this paper, a number of classification methods, including XGBoost, SVM, RF, and DNN, have been used. It should be noted that other classifiers, including k-nearest neighbors (KNN) and naïve Bayes classifiers, have also been assessed empirically; however, they were not reported due to their low performance. In order to assess various classifiers, they have been compared in terms of various metrics, including accuracy, error rate, etc. The results are reported in the following.

Parameter tuning is performed based on a series of trials. A penalty factor of 2 along with the RBF kernels are used in SVM classification. The number of random trees in the RF classifier has been 100, with the Gini split criterion. An exponential cost function is used in XGBoost. The number of estimators and the learning rate have been 80 and 0.1, respectively. Also, the DNN is modeled by a 4-layered structure with a learning rate of 0.08.

Moreover, as shown in Table 3, the feature selection method has increased the performance of various classifiers. Indeed, the highly discriminative features have been selected via the F-score method, and redundant ones have been eliminated. This task has improved the performance measures of all of the approaches. Regarding the different dimensionality of datasets, a variety of features have been selected based on a number of trials. Apparently, no unique combination outperforms the others in all of the datasets globally. In *H. sapiens* and *M. musculus*, the second combination has better performance, whilst the third is the best for *E. coli.* Regarding the number of training samples and the structural differences between protein sequences across the datasets, the extracted features have different discrimination performances for each dataset, and they would differ. By eliminating the redundant and uncorrelated features at the phase of feature selection, the second combination outperforms the others in all of the datasets.

**Table 3:** Classification performance with the combination of features when F-score is applied for feature selection.

Dataset	Combination	Classifier	Acc(%)	Sn (%)	Sp (%)	MCC	AUC
*E. coli*	TF-CRF, EGAAC, TF-IDF	SVM	64.84	66.12	57.13	0.7123	0.7682
		RF	92.78	93.76	94.18	0.8934	0.9734
		XGBoost	95.93	93.24	95.89	0.9087	0.9867
		DNN	72.07	76.92	67.15	0.7584	0.8241
	EAAC,PKA, PSSM, TF-CRF	SVM	71.39	71.87	70.91	0.8411	0.8923
		RF	97.17	94.67	95.87	0.9265	0.9761
		XGBoost	97.65	**95.71**	**96.29**	0.9328	**0.9846**
		DNN	95.18	97.11	96.06	0.9261	0.9704
	EAAC, PKA, PSSM, TF-CRF, EGAAC	SVM	68.56	71.67	71.46	0.8663	0.9037
		RF	96.92	93.88	96.21	0.9241	0.9769
		XGBoost	**97.67**	94.79	95.91	**0.9378**	0.9821
		DNN	93.12	99.34	85.73	0.9023	0.9625
*H. sapiens*	TF-CRF, EGAAC, TF-IDF	SVM	67.21	69.34	68.84	0.7523	0.8934
		RF	92.94	91.88	93.79	0.9123	0.9517
		XGBoost	94.18	96.18	95.08	0.9234	0.9634
		DNN	80.23	87.53	71.32	0.8032	0.9137
	EAAC,PKA, PSSM, TF-CRF	SVM	71.97	73.15	71.20	0.8769	0.9022
		RF	94.45	93.41	95.32	0.9327	0.9618
		XGBoost	**96.32**	**98.11**	**97.89**	**0.9541**	**0.9822**
		DNN	92.41	95.23	95.39	0.9243	0.9411
	EAAC, PKA, PSSM, TF-CRF, EGAAC	SVM	73.12	62.8	73.42	0.8671	0.9038
		RF	93.21	93.44	95.15	0.9128	0.9593
		XGBoost	95.72	97.23	97.31	0.9483	0.9695
		DNN	92.37	93.89	93.72	0.9134	0.9609
*M. muculus*	TF-CRF, EGAAC, TF-IDF	SVM	65.91	65.58	66.23	0.8109	0.9037
		RF	92.55	93.21	93.88	0.9023	0.9618
		XGBoost	93.54	95.77	94.54	0.9142	0.9765
		DNN	73.41	90.59	58.72	0.8517	0.9132
	EAAC,PKA, PSSM, TF-CRF	SVM	77.79	77.5	78.07	0.8923	0.9129
		RF	93.78	94.78	95.46	0.915	0.9678
		XGBoost	**94.78**	**96.88**	**96.75**	**0.9356**	**0.9778**
		DNN	91.55	93.43	94.89	0.9095	0.9508
	EAAC, PKA, PSSM, TF-CRF, EGAAC	SVM	73.32	74.41	75.21	0.8871	0.9173
		RF	92.89	94.67	94.89	0.9097	0.9694
		XGBoost	94.13	96.45	95.72	0.9254	0.9743
		DNN	92.26	94.28	94.51	0.8156	0.9516

Significant values are in bold.

As depicted in Fig. 4, TFCRF has shown the best performance in all of the datasets. In this scheme, weighting features is performed by considering their distribution in classes, in addition to their distribution in sequences. Also, the weighting has not been independent of the number of sequences in each class. This issue has increased the classification performance based on TFCRF. In comparison with other feature weighting schemes, this method can drastically increase classification performance.

**Figure 5:**
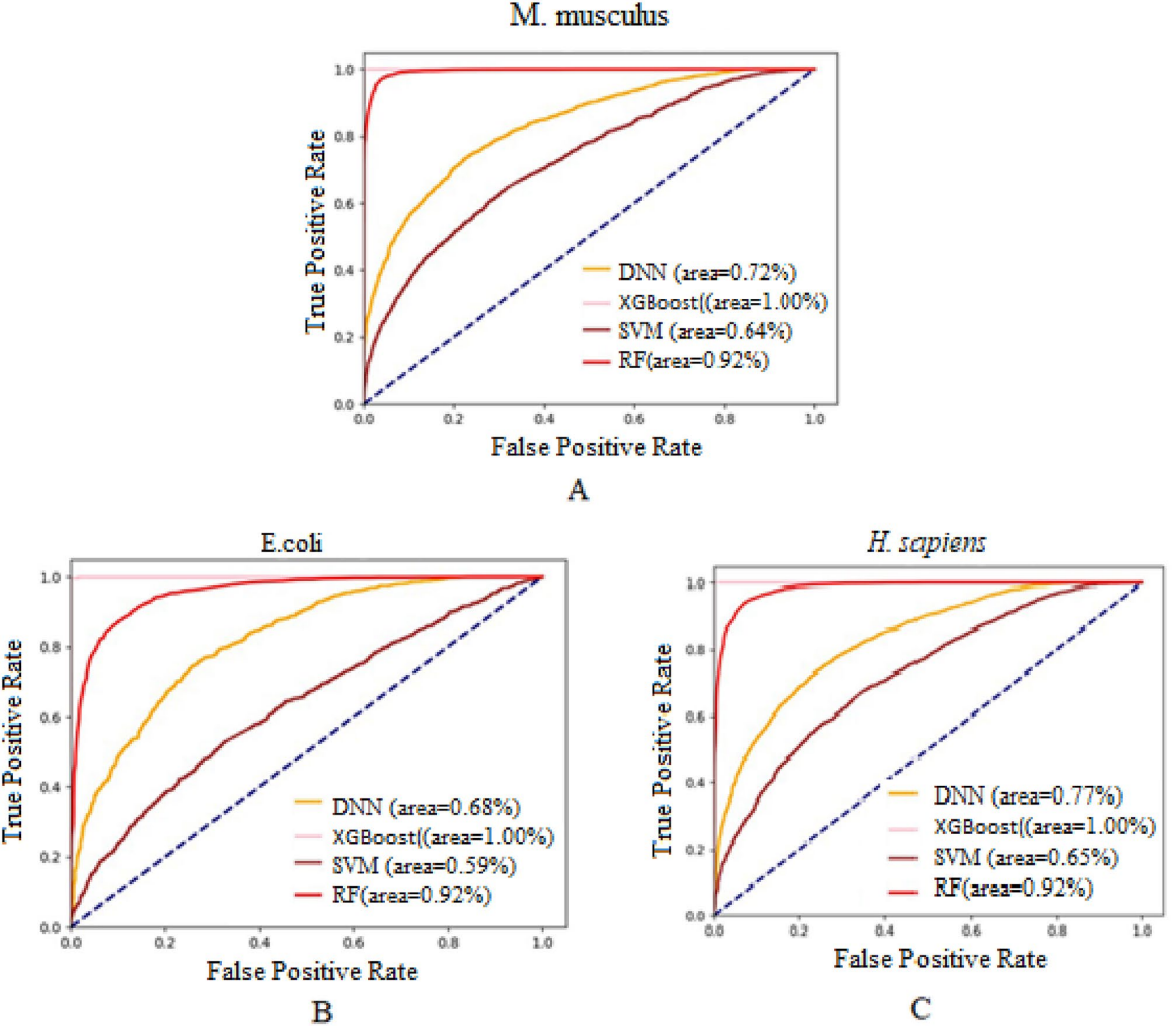
The ROC curve for the proposed method. (**A**), (**B**) and (**C**) diagrams pertain to *M. musculus*, *E. coli*, and *H. sapiens* datasets, respectively.

In order to deeper analysis of various feature combinations, the ROC diagram on the training dataset is sketched in Fig. 5. The ROC curve is depicted for the third combination, and selecting 80% of the best features in the datasets *M. musculus*, *E. coli*, and *H. sapiens*. As evident in the ROC curve of SVM, XGboos, RF, and DNN classifiers, the area under the curve for XGboost is considerably greater than that of the other methods, indicating its potent generalization and high performance for malonylation and non-malonylation site prediction of lysine proteins.

The values of AUPR and AUROC for various classifiers on the three datasets are tabulated in Table 4. As it can be seen, XGBoost outperforms the other methods. To study the significance of the results, the p-values of AUPR (namely P-AUPR) and AUROC (namely P-AUROC) for various methods and datasets are depicted in Table 4 too. As it can be seen, the prediction rate of each method is significantly higher than that of random prediction. In addition, XGBoost classifier outperforms the others, having a lower P-value.

**Table 4:** The values of AUROC, AUPR and their P-values for various classifiers and datasets.

Method	*E.coli*				*H. sapiens*				*M. muculus*			
	AUPR	AUROC	P-AUPR	P- AUROC	AUPR	AUROC	P-AUPR	P- AUROC	AUPR	AUROC	P-AUPR	P- AUROC
SVM	0.122	0.59	9.47E−2	7.73E−02	0.141	0.65	1.00E−11	1.86E−07	0.136	0.64	1.09E−9	1.83E−6
RF	0.193	0.92	2.46E−37	7.00E−18	0.201	0.92	1.45E−71	6.83E−21	0.198	0.92	2.38E−58	5.34E−20
XGBoost	0.291	1.00	1.76E−46	1.20E−19	0.311	1.00	6.59E−76	3.16E−31	0.304	1.00	6.59E−68	3.16E−28
DNN	0.134	0.68	5.62E−05	7.98E−07	0.157	0.77	3.16E−31	1.27E−13	0.154	0.72	2.75E−27	1.36E−11

**Figure 6:**
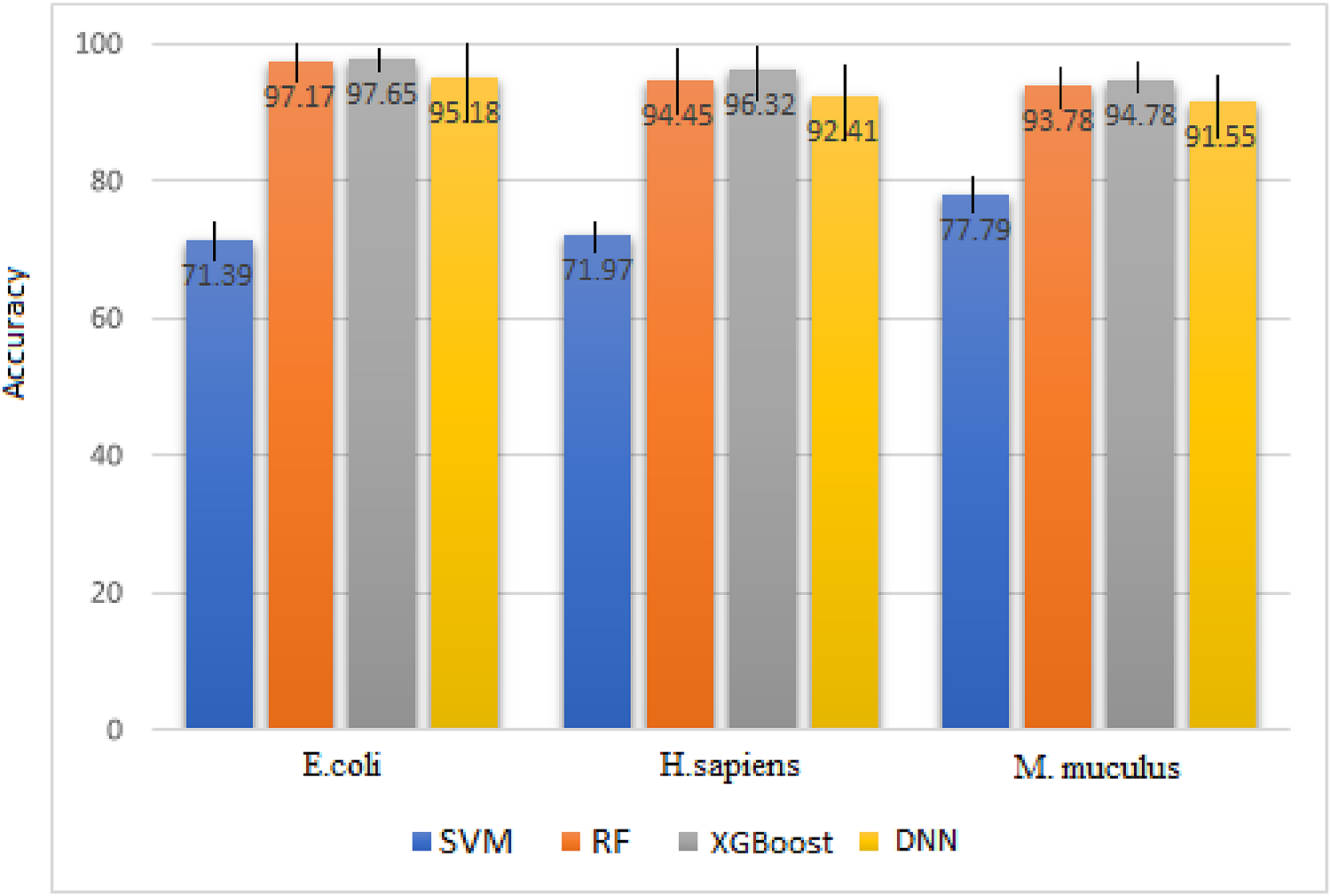
Studying classification models based on error bars for *E. coli*, *M. musculus*, and *H. sapiens*.

Error analysis is carried out to depict model resistivity and stability. The error bar conveys estimated errors or uncertainty in order to achieve a deeper understanding of the measurements. Typically, error bars are used to denote the standard deviation, standard error, confidence intervals, or minimum/maximum values in a dataset. The length of an error bar helps to picture the uncertainty associated with a data point. A short error bar shows the compaction of values, signaling that the mean value has had a further effect in the training model, whilst a long error bar addresses sparsity and a lesser number of data values. A comparison is carried out between DNN, RF, XGBoost, and SVM. The accuracies of the algorithms via a tenfold cross-validation strategy are pictured out in Fig. 6 for the underlying datasets. As evident from Fig. 6, XGBoost has outperformed the others, and DNN depicts the highest error regarding the lengths of the bars. The lesser length of the error bars in Fig. 6 states a higher accuracy of the due algorithm and lower variance of the model accuracy. According to this diagram, it can be concluded that the results of iterations in the tenfold cross-validation have been close in XGBoost, leading to errors approximately equal to zero. Therefore, this model has a high generalization performance. However, the reverse has taken place for DNN, addressing that the results of the iterations in tenfold cross-validation are not close, leading to a higher variance in the accuracy, and hence, a lower generalization performance.

### Evaluation through comparison with other methods

In order to further analysis, the proposed method is compared with various prediction methods for the datasets *E. coli*, *H. sapiens*, and *M. musculus* in terms of ACC, SN, SP, and MCC measures. The results are taken in Table 5. As shown, the proposed method has outperformed Malopred^11^, kmal-sp^14^, DeepMal^28^ and RF-MaloSite^40^ with a higher ACC, SN, SP, and MCC, in all of the datasets. The 97.21% accuracy of the proposed method for *E. coli* is 12.71%, 17.41%, 4.2% greater than kmal-sp, MaloPred, and DeepMal, respectively. The 95.22% ACC index of the proposed method for *H. sapiens* is also 4.3% to 20.22% greater than the other prediction models. Performance measures MCC and AUC are high for this dataset too. The 94.31% accuracy of the proposed method for *M. musculus* is greater than the other prediction approaches. The 92.17% MCC of the proposed method outperforms the others for this dataset and has considerably improved the results for malonylation site prediction.

**Table 5:** A comparison between the proposed method and the approaches of DeepMal, Kmal-sp, Malopred, and RF-MaloSite.

Dataset	Methods	Acc(%)	Sn (%)	Sp (%)	MCC	AUC
*E. coil*	Deepmal	93.01	91.71	94.31	0.8607	0.951
	Malopred	79.8	75.0	81.0	0.561	0.755
	Kmal-sp	84.5	0.83	0.86	0.69	0.930
	RF-MaloSite	–	–	–	–	–
	Proposed method	97.21	94.31	95.72	0.9279	0.9768
*H. sapiens*	Deepmal	90.92	91.61	90.22	0.8186	0.9447
	Malopred	82.7	82.9	82.4	0.653	0.871
	Kmal-sp	86.0	84.9	87.0	0.720	0.944
	RF-MaloSite	75	84	65	0.50	0.78
	Proposed method	95.22	97.32	97.14	0.9448	0.9749
*M. musculus*	Deepmal	91.93	92.3	91.57	0.8045	0.9534
	Malopred	78	91.71	94.31	0.8607	0.827
	Kmal-sp	83.3	82.9	83.7	0.667	0.923
	RF-MaloSite	68	72	65	0.36	0.75
	Proposed method	94.31	96.47	95.34	0.9217	0.9721

Since the extracted features are based on TFCRF in the proposed scheme, the discrimination performance is higher (as discussed in Sections “Feature extraction” to “Term frequency and category relevancy factor (TF-CRF)”); thus, a higher recognition rate is achieved. In addition, dimension reduction through selecting highly relevant features has increased the performance of the proposed method since model overfitting is potentially mitigated.

## Conclusion

In this paper, a machine learning-based method has been proposed for malonylation site prediction. Since the input features are crucial in machine-learning models, several features, including a novel one based on TF-CRF, have been extracted out of protein sequences. Next, the features are combined. Since feature combination leads to high dimensional data and, in turn, model overfitting, the most efficient and discriminating features have been chosen based on a feature selection method. The results show that XGboost outperforms the other classifiers based on the extracted and selected features.

## Data Availability

The datasets used and/or analyzed during the current study are available from the corresponding author on reasonable request.
